# Daikenchuto increases blood flow in the superior mesenteric artery in humans: A comparison study between four-dimensional phase-contrast vastly undersampled isotropic projection reconstruction magnetic resonance imaging and Doppler ultrasound

**DOI:** 10.1371/journal.pone.0245878

**Published:** 2021-01-27

**Authors:** Katsunori Suzuki, Yasuo Takehara, Mayu Sakata, Masanori Kawate, Naoki Ohishi, Kosuke Sugiyama, Toshiya Akai, Yuhi Suzuki, Masataka Sugiyama, Takafumi Kawamura, Yoshifumi Morita, Hirotoshi Kikuchi, Yoshihiro Hiramatsu, Masayoshi Yamamoto, Hatsuko Nasu, Kevin Johnson, Oliver Wieben, Kiyotaka Kurachi, Hiroya Takeuchi

**Affiliations:** 1 Second Department of Surgery, Hamamatsu University School of Medicine, Hamamatsu, Shizuoka, Japan; 2 Department of Fundamental Development for Advanced Low Invasive Diagnostic Imaging, Nagoya University, Graduate School of Medicine, Nagoya, Aichi, Japan; 3 Department of Radiology, Hamamatsu University Hospital, Hamamatsu, Shizuoka, Japan; 4 Department of Diagnostic Radiology & Nuclear Medicine, Hamamatsu University School of Medicine, Hamamatsu, Shizuoka, Japan; 5 Department of Medical Physics, University of Wisconsin, Madison, WI, United States of America; 6 Department of Radiology, University of Wisconsin, Madison, WI, United States of America; The Ohio State University, UNITED STATES

## Abstract

Respiratory-gated four-dimensional phase-contrast vastly undersampled isotropic projection reconstruction (4D PC-VIPR) is magnetic resonance (MR) imaging technique that enables analysis of vascular morphology and hemodynamics in a single examination using cardiac phase resolved 3D phase-contrast magnetic resonance imaging. The present study aimed to assess the usefulness of 4D PC-VIPR for the superior mesenteric artery (SMA) flowmetry before and after flow increase was induced by the herbal medicine Daikenchuto (TJ-100) by comparing it with Doppler ultrasound (DUS) as a current standard. Twenty healthy volunteers were enrolled in this prospective single-arm study. The peak cross-sectionally averaged velocity was measured by 4D PC-VIPR, peak velocity was measured by DUS, and flow volume (FV) of SMA and aorta were measured by 4D PC-VIPR and DUS 25 min before and after the peroral administration of TJ-100. The peak cross-sectionally averaged velocity, peak velocity, and FV of SMA measured by 4D PC-VIPR and DUS significantly increased after administration of TJ-100 (4D PC-VIPR: the peak cross-sectionally averaged velocity; *p* = 0.004, FV; *p* = 0.035, DUS: the peak velocity; *p* = 0.003, FV; *p* = 0.010). Furthermore, 4D PC-VIPR can analyze multiple blood vessels simultaneously. The ratio of the SMA FV to the aorta, before and after oral administration on the 4D PC-VIPR test also increased (*p* = 0.015). The rate of change assessed by 4D PC-VIPR and DUS were significantly correlated (the peak cross-sectionally averaged velocity and peak velocity: r = 0.650; *p* = 0.005, FV: r = 0.659; *p* = 0.004). Retrospective 4D PC-VIPR was a useful modality for morphological and hemodynamic analysis of SMA.

## Introduction

Gastrointestinal blood flow changes dynamically in response to various factors, such as diet, exercise, body temperature, medicine, and inflammation. Currently, computed tomography angiography has been used to assess the intestinal arterial morphology [1], and Doppler ultrasound (DUS) flowmeters have been used to quantify intestinal blood flow [2,3]. Contrast-enhanced computed tomography is useful for consistent evaluation of the vascular network, but it cannot provide blood flow information, such as flow direction or velocity. DUS is a convenient non-invasive technique that is often preferred for measuring flow direction, volume, and velocity [4]. However, DUS is not always reproducible because it depends on the operator’s skill. Furthermore, DUS is often hindered by problematic overlaps of the gastrointestinal gas interference. Thus, an alternative robust technique that can image and quantify the blood flow in one examination would be useful.

Respiratory-gated four-dimensional (4D) phase-contrast vastly undersampled isotropic projection reconstruction magnetic resonance imaging (MRI) (4D PC-VIPR) is a MR technique that can provide retrospective information on vascular morphology and hemodynamics in a single examination [5,6]. Without using any contrast agent, objective and quantitative analysis of the flow direction and velocity of any blood vessel within the field of view (FOV) can be performed by 4D PC-VIPR. 4D PC-VIPR uses a non-Cartesian κ-space trajectory similar to a radial scan where all data lines traverse the center of κ-space, which is different from other MR angiography (MRA) using Cartesian sampling. The image contrast provided by 4D PC-VIPR is higher, because the echoes include highest signals at the center of κ-space. For measurement of plane determinations on 4D PC-VIPR, MR angiography using magnitude images and streamline cine images within the lumen were postprocessed. Unlike DUS, 4D PC-VIPR enables retrospective flowmetry of vessels with different measurement planes while avoiding non-laminar flow. Previous studies using 4D PC-VIPR initially applied it to imaging of intracranial vessels [7–9], cardiac, and thoracic vessels [10,11]. Recently, this MRI technique was used to assess the renal artery before and after percutaneous transluminal angioplasty for patients with renal hypertension [12] and to assess renal transplantation [13]. However, there are few reports on blood flowmetry for the superior mesenteric artery (SMA).

TJ-100 (Daikenchuto) is a well-established traditional Japanese herbal medicine (Kampo) effective for improving gastrointestinal motility and preventing postoperative adhesional and paralytic ileus after abdominal surgery [14–18]. Previous studies have shown that TJ-100 induces adrenomedullin (ADM) release from intestinal epithelium and induces calcitonin gene-related peptide (CGRP) from the nerve endings of the vascular smooth muscle to increase intestinal blood flow, agonized by transient receptor potential A1 (TRPA1) and transient receptor potential V1 (TRPV1). [19,20]. Moreover, TJ-100 enhanced the intestinal motility. TJ-100 exerts an acetylcholine-releasing action on the smooth muscle tissue of the ileum via 5-HT3 and 5-HT4 receptors; promotes the secretion of motilin, a gastrointestinal hormone that improves intestinal motility; and leads to the release of substance P by TRPV1 stimulation [21–24]. In addition, TJ-100 has anti-inflammatory effect via ADM and cyclooxygenase-2 (COX-2) [25,26]. These effects have been shown to be clinically effective for improving small bowel motility [27,28] and to increase colonic blood flow in animal models [29]. After that, TJ-100 has been reported to increase SMA blood flow evaluated by DUS in humans [4].

The aim of this study is to compare the usefulness of 4D PC-VIPR SMA flowmetry before and after the flow increase induced by the herbal medicine Daikenchuto (TJ-100) with the current standard of conventional DUS.

## Materials and methods

### Subjects

The protocol of this single-arm prospective study was approved by the Ethics Committee of Hamamatsu University School of Medicine (Institutional Review Board approval number of Hamamatsu University School of Medicine: # E17-027). Participants were recruited by flyers posted around our university. Subjects for whom MRI was contraindicated who had previous abdominal surgery, who were taking oral medicine or supplements, or who were pregnant or lactating were excluded.

Twenty healthy subjects aged ≥20 years with systolic blood pressure <140 mmHg were recruited and enrolled in this study between May and December 2017. Written informed consent to participate was obtained from all subjects prior to their participation.

### Methods

**Fig 1:**
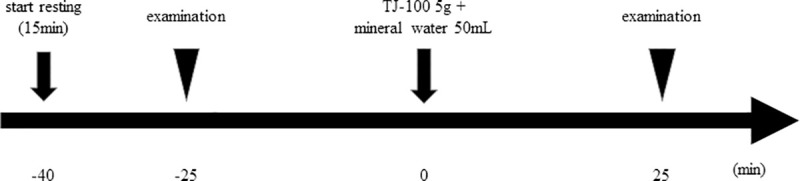
Outline of the study. The subjects were examined after ≥2-hour fasting. After 15 min of rest, the first examination was performed. After 25 min, 5.0 g of TJ-100 and 50 ml of mineral water were administered orally followed by 25 min of rest by the subject, and then the secondary examination was performed. All participants underwent both DUS and PC-VIPR within 4 weeks, with a minimum interval of 1 week.

Timeline of this study is shown in Fig 1. All participants were fasted for at least 2 hours before study entry. The first examination was performed after 15 min of rest to avoid any effects of exercise. Twenty-five minutes after the baseline flowmetry, 5.0 g of TJ-100 (Tsumura, Co., Tokyo, Japan) with 50 ml of mineral water was orally administered. Then, rest again for 25 min, and the second examination was performed (Fig 1). Each participant underwent the first examinations within 4 weeks after enrollment. All participants underwent both DUS and 4D PC-VIPR within 4 weeks, with a minimum interval of 1 week.

### DUS

**Fig 2:**
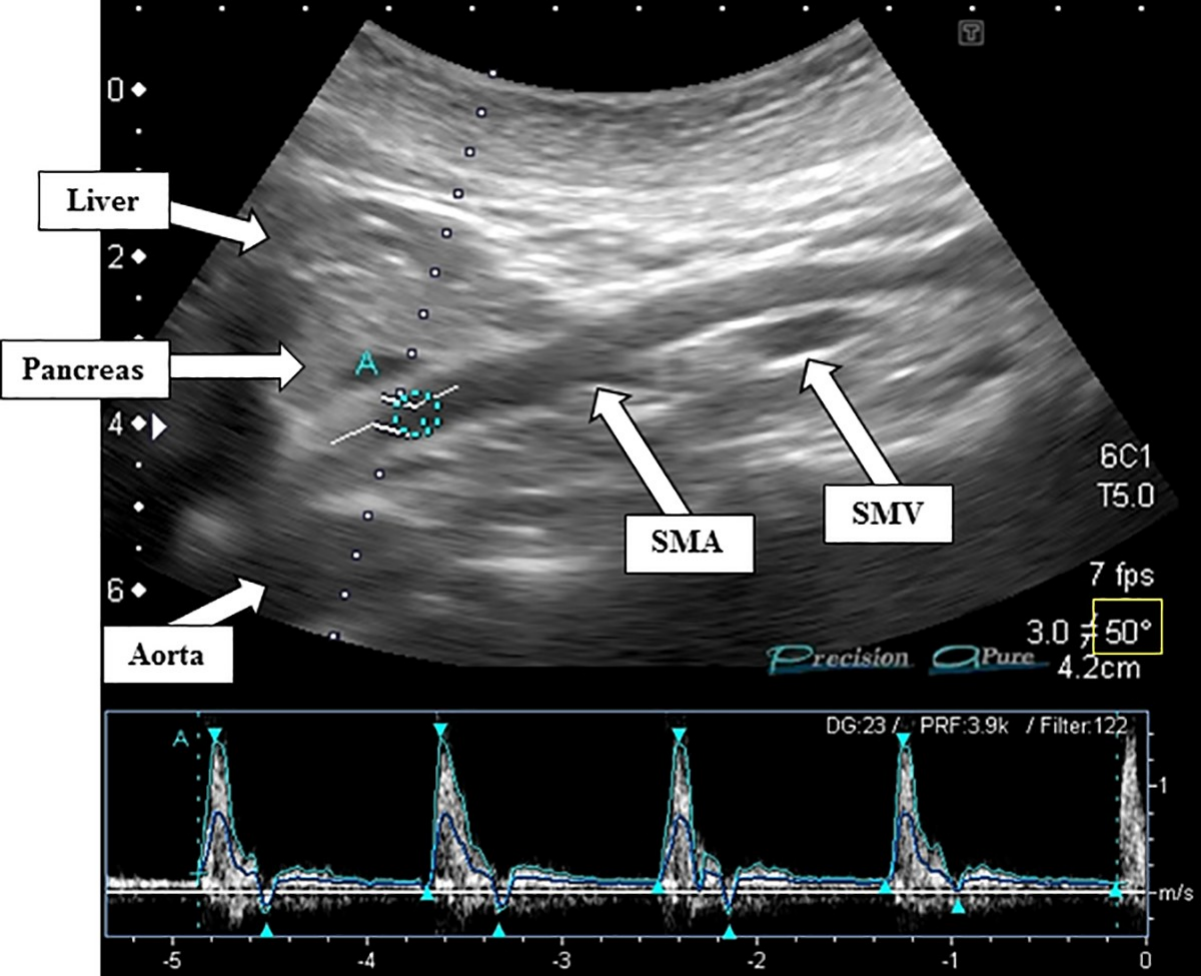
Measurement of SMA blood flow by DUS. Blood flow velocity was measured 3 cm from the origin of the SMA by pulsed DUS. The waves highlighted in light blue show four cardiac cycles (bottom).

The DUS device used in this study was a Xario SSA-660A (Canon Medical Systems Corp., Tochigi, Japan). The scans were performed by an experienced operator with an experience of 23 years. The measurement was performed in the consultation room and the room temperature was adjusted to 24°C–25°C. The cross-sectional area of the SMA was calculated by measuring the inner diameter of the vessel. Pulsed Doppler signals were acquired within 2–3 cm of the origin of the artery [30,31]. For accurate measurement, a Doppler angle of ≤60° was used [4,32]. Each Doppler waveform was drawn automatically and calculated by using the software in the US system (FlowVol in L/min) = Vm_peak (cm/s) × 60 (s/min) × Area (mm^2^) / 100 / 1000). The flow volume (FV) was calculated as the vessel cross-sectional area multiplied by the mean blood flow velocity [31]. The FV was recorded in four cardiac cycles (Fig 2). The examination time of the Doppler measurement was approximately 2–3 min.

### Noncontrast MRA and SMA flowmetry using 4D PC-VIPR

All MR studies were conducted using a 3.0T MR scanner (Discovery 750, GE Healthcare, Milwaukee, WI) together with a 32-channel torso phased-array torso coil (GE Healthcare). 4D PC-VIPR was performed in the MRI room of our hospital. The room temperature was adjusted to 21°C–22°C. For estimating the approximate flow velocity of the SMA, 2D cine PC was performed prior to 4D PC-VIPR. The velocity encoding values (VENC) for 4D PC-VIPR were determined based on the values. To avoid aliasing, optimized VENC was determined individually as the value that was 30% greater than the maximum velocities measured with 2D cine PC. The measuring planes for 2D cine PC were placed perpendicular to the SMA at the proximal portion, and then 4D PC-VIPR was performed. ECG-gated 4D PC-VIPR that was conducted covering the SMA segment ranging from the diaphragm and bilateral main renal arteries. A fast spoiled gradient echo-based 4D PC-VIPR was operated using parameters as follows: TR (repetition time) / TE (echo time) / FA (flip angle) of 7.0–7.1 ms / 3.3–3.4 ms /8 degree, FOV of 32 cm, slice thickness of 1.25 mm, locs/slab of 8, matrix of 256 × 256, receiver bandwidth of 62.5 kHz, partition thickness of 1.25 mm, NEX (number of excitations) of 1, number of phases/location of 20, imaging time of 9:29–9:36 min, respiratory window of 50%, number of projections of 10,000, and the velocity encoding (VENC) depending on the +30% of the maximum velocities measured in the preceding 2D cine PC studies. The trajectory of the data filling was non-Cartesian radial sampling. The examination takes approximately 2–3 min to set up; the 2D cine PC and measurements take approximately 2–3 min, and the total examination lasts for approximately 15 min.

### Image reconstruction, DICOM data conversion, and postprocess

Acquired data were reconstructed on an offline workstation. The reconstructed binary data was converted to DICOM format, and the phase and magnitude data for each x, y, z axes were then transferred to a personal computer installed with dedicated flow analysis software (flova II; R’s Tech, Hamamatsu, Japan). First, the time-averaged magnitude data was postprocessed to create MRA using a maximum intensity projection algorithm. The 3D flow information was interpolated with the MRA spatial data with resolution of 1.2 × 1.2 × 1.2 mm.

### Analysis of flow dynamics with 3D Cine PC

**Fig 3:**
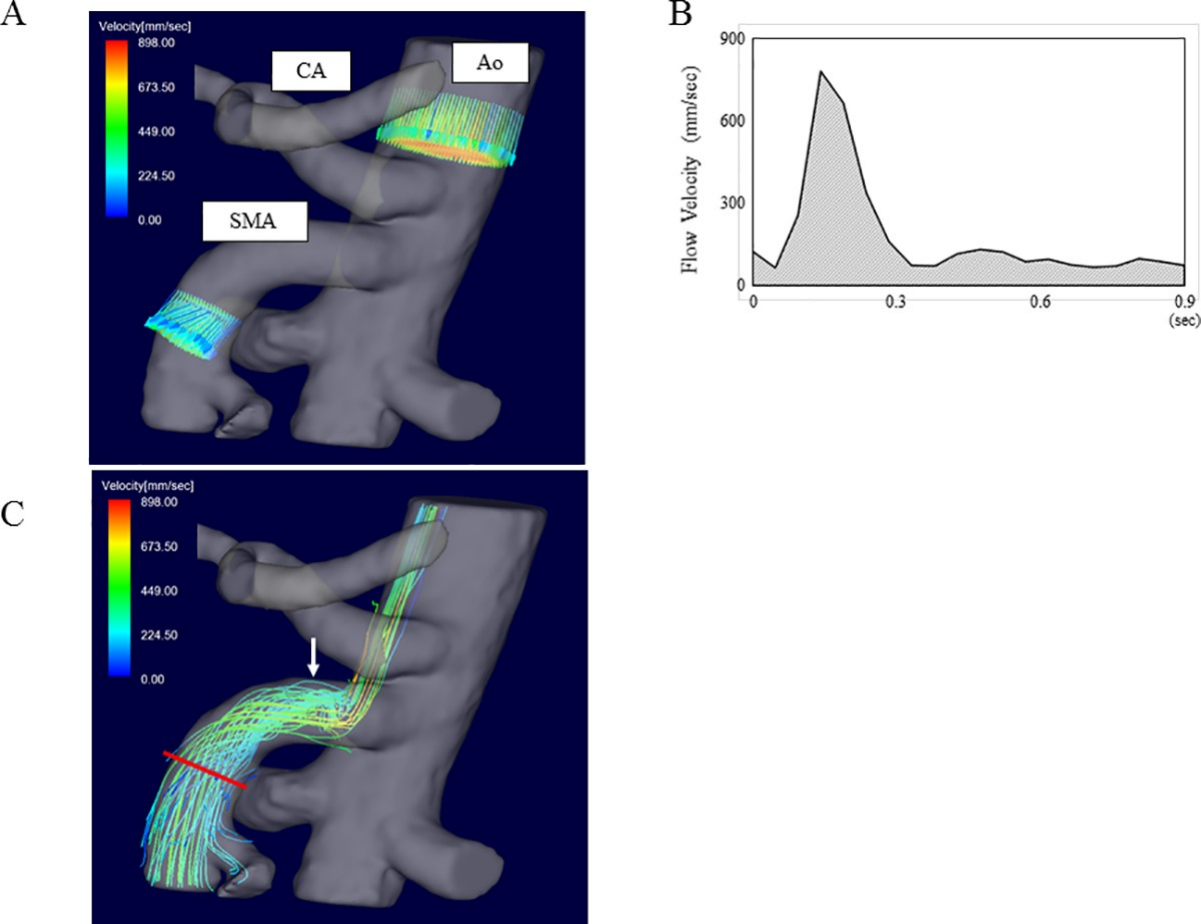
Measurement of SMA blood flow by 4D PC-VIPR. (A) Abdominal blood vessels depicted by 4D PC-VIPR. Three-dimensional velocity data derived from 4D PC-VIPR was incorporated with the MRA morphology in a 3D vector field fashion. Flow velocity and FV can be measured at any location analyzed by using dedicated software. (B) Graph of the values obtained from the data. The shaded area indicates FV. (C) Flow velocity can be measured in all locations of the SMA by adding the blood flow velocity and direction as vector data using dedicated software. Disturbed non-laminar (i.e., vortex and/or helical) flow (arrow) can be shown by streamline analysis. The measurement was performed while avoiding the area of non-laminar flow.

Datasets were analyzed with the flow analysis software flova. The 3D vector field, streamlines, and pathlines were delineated using the Runge–Kutta method. The flow velocity was measured on the perpendicular sections determined on the SMA and the aorta (Fig 3A). The data obtained from the area was converted into a graph, the cardiac cycle was visualized, and the area under the curve was obtained as a FV (Fig 3B).

### Analysis of the streamline

Since SMA velocimetry was dependent on the localization of the measurement planes [33], streamline images were created by 4D PC-VIPR, and the optimal plane was selected to avoid disturbing the flow (Fig 3C).

### Reagents

TJ-100 (Tsumura Co., Tokyo, Japan; Standard Commodity Classification No. of Japan: 875200, Approval No.: (61AM)3299) was obtained in the form of a dried powder, which was manufactured as an aqueous extract containing processed ginger (*Zingiber officinale*), ginseng (*Panax ginseng*), and zanthoxylum fruit (*Zanthoxylum piperitum*) at a ratio of 5:3:2. These plants are all registered in the Pharmacopoeia of Japan. The three herbal medicines were extracted with purified water at 95°C for 1 h and then spray-drying was used to produce a powder. TJ-100 was made by mixing Daikenchuto extract powder and maltose syrup powder (Koi) at a ratio of 1:8 [34]. It is a Kampo extract formulation that is made into granules using Tsumura's original dry granulation method that does not use any organic solvent or water. This study was performed using 5.0 g of TUMURA Daikenchuto Extract Granules for Ethical Use (lot number: L46022). Research has shown that 5.0 g of TJ-100 administration successfully increased the SMA blood flow in 20 min and 40 min, as assessed using DUS [4]; further, a dose of 5.0 g has been approved as a single dose for daily medical care (http://mpdb.nibiohn.go.jp/stork/). The confirmation test method for the active ingredient in the drug product was thin-layer chromatography as described by Tsumura. The dosage form is granules; the color is light grayish white, the smell is characteristic smell, and the taste is sweet and pungent.

### Statistical analysis

The peak velocity measured with DUS, peak cross-sectionally averaged velocity measured with 4D PC-VIPR, FV measured by 4D PC-VIPR and DUS, and the ratio of the SMA FV to the aorta FV measured using 4D PC-VIPR before and after TJ-100 administration were compared by using the Wilcoxon signed-rank test. The non-parametric Spearman’s rank correlation coefficient was used in analysis of the correlation between the rate of change of the peak velocity and peak cross-sectionally averaged velocity, and both FV. With respect to sample size determination, the sample size of the Wilcoxon signed-rank test (matched pairs) was calculated as follows: d = 0.8, α = 0.05 and 1 − β = 0.8. As per these parameters, the sample size was n = 15. A review of previous similar studies [4,14,15,18,27,35,36] showed that 10–19 cases were sufficient to determine the significance of the blood flow in the SMA following the administration of the TJ-100. Therefore, the target number of cases was determined as 20. The sample size was calculated using G*power 3.1.9.7 [37,38]. Statistical analyses were performed using SPSS software (version 25.0, SPSS Japan Inc., Tokyo, Japan). The Bland–Altman plot was used for analyzing the agreement between the two different techniques. It was used to show that there was only a random difference and no systematic differences. *p–*values < 0.05 were considered to be indicative of statistical significance.

## Results

The mean age of the subjects was 27.9 years (range: 20–56 years), with 12 males and 8 females. The characteristics of the subjects are shown in Table 1.

**Table 1:** Characteristics of the 20 healthy subjects in this study.

Characteristics of the subjects (n = 20)	
**Age (years)**	27.9 ± 9.0
**Height (cm)**	166.7 ± 7.1
**Weight (kg)**	59.1 ± 7.8
**Body mass index (kg/m**^**2**^**)**	21.2 ± 2.1
**Sex (Male:Female ratio)**	12:8

Data was presented as mean ± standard deviation (SD).

The details of environmental and patients conditions are shown in S1 Table. The interval between the two examinations was 1 to 4 weeks. No significant difference between DUS and 4D PC-VIPR was found for fasting duration (*p* = 0.808) and body temperature (*p* = 0.460). In DUS, SMA flowmetry was feasible in all 20 subjects. 4D PC-VIPR was successfully performed in all 20 subjects; however, flowmetry was not feasible in three cases, owing to the overflow of stored data of the MRI in two cases and the uncommon vessel anatomy (the right hepatic artery branching from the SMA) in one case.

**Fig 4:**
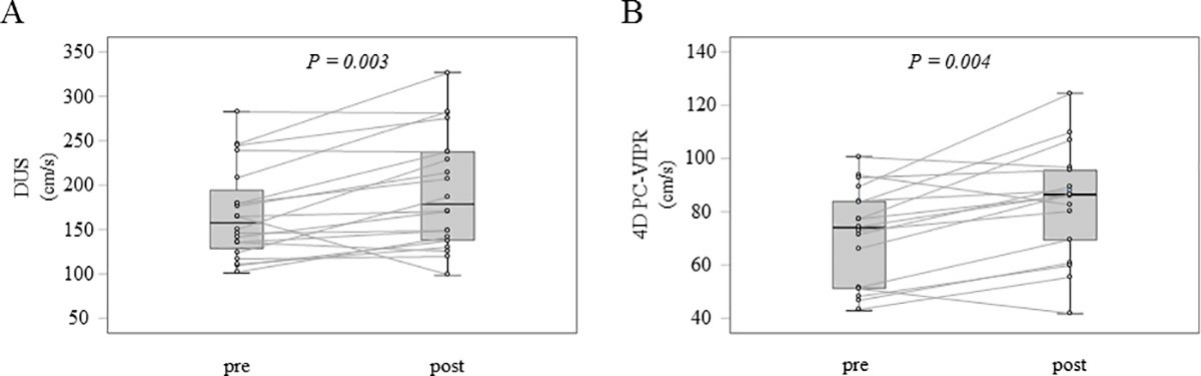
Peak velocity and peak cross-sectionally averaged velocity of SMA. (A) Comparison by actual measurement before and after oral administration in DUS (*p* = 0.003, Wilcoxon signed-rank test). (B) Comparison of measured values before and after oral administration in 4D PC-VIPR (*p* = 0.004, Wilcoxon signed-rank test).

The peak velocity of SMA measured with DUS (Fig 4A) and peak cross-sectionally averaged velocity of SMA measured with 4D PC-VIPR (Fig 4B) were significantly increased after TJ-100 administration. The rates of change before and after administration were 16.5 ± 4.8% increase on DUS (*p* = 0.006) and 24 ± 4.2% increase on 4D PC-VIPR (*p* = 0.004; median ± standard error (SE)).

**Fig 5:**
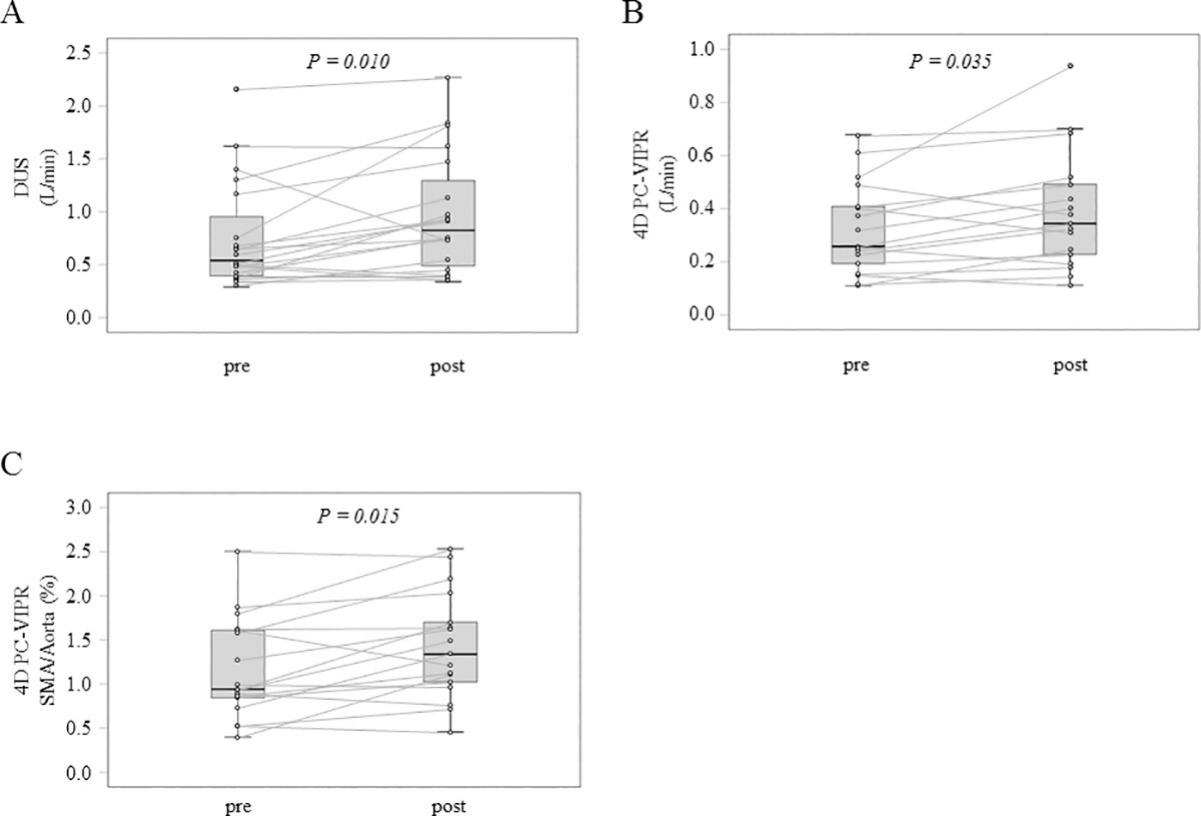
Blood flow volume of SMA. (A) Comparison by actual measurement before and after oral administration in DUS (*p* = 0.010, Wilcoxon signed-rank test). (B) Comparison by actual measurement before and after oral administration in 4D PC-VIPR test (*p* = 0.035, Wilcoxon signed-rank test). (C) The ratio of the SMA FV to the aorta, before and after oral administration on 4D PC-VIPR test (*p* = 0.015, Wilcoxon signed-rank test).

The mean FV was also significantly increased after TJ-100 administration on DUS (Fig 5A) and on 4D PC-VIPR (Fig 5B). The median rates of change were 31.5 ± 11.8% increase on DUS (*p* = 0.015) and 21 ± 9.4% increase on 4D PC-VIPR (*p* = 0.039; median ± SE).

Because 4D PC-VIPR can analyze by excising an arbitrary cross section, analyzing multiple blood vessels simultaneously is possible. Therefore, we also evaluated the ratio of the SMA FV to the aorta using 4D PC-VIPR to confirm that the results were not towing to an increase in the cardiac output. We found that the SMA/aorta FV ratio was also significantly increased by TJ-100 administration (Fig 5C).

**Fig 6:**
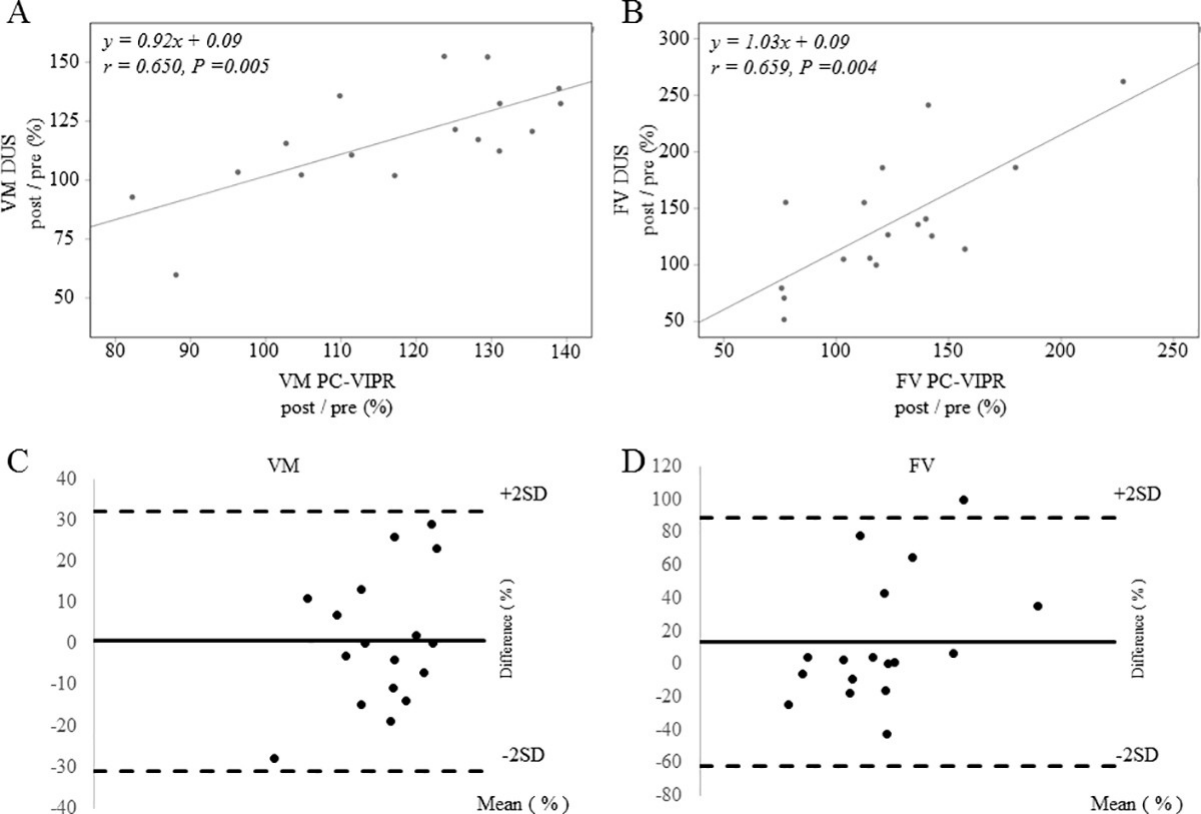
Correlation between the rates of change on DUS and 4D PC-VIPR. (A) Correlation between the rates of change on DUS and on 4D PC-VIPR at the peak velocity and peak cross-sectionally averaged velocity of the SMA (r = 0.650; *p* = 0.005, Spearman’s rank correlation coefficient). (B) Correlation between the rates of change on DUS and on 4D PC-VIPR in the blood FV of the SMA (r = 0.659; *p* = 0.004, Spearman’s rank correlation coefficient). Bland–Altman plots of the change rates before and after oral administration (post/pre) measured by 4D PC-VIPR and DUS for (C) the peak velocity and peak cross-sectionally averaged velocity and (D) blood FV. The y-axis shows the difference between the two measurements, and their mean is shown on the x-axis. The continuous line represents the bias; i.e., the mean of the differences, whereas the dotted lines indicate the limits of agreement (bias ± 2 SD).

The correlation between the change rate of the peak velocity and peak cross-sectionally averaged velocity and blood flow of SMA was statistically significant in both 4D PC-VIPR and DUS measurement, with the regression coefficients of the peak velocity and peak cross-sectionally averaged velocity: r = 0.650 (*p* = 0.005), FV: r = 0.659 (*p* = 0.004), respectively. Measured bias between the two modalities was within the acceptable range (Fig 6).

## Discussion

In this study, we demonstrated that 4D PC-VIPR could assess SMA blood flow without the use of contrast agents, and the accuracy was comparable to that of conventional DUS. Furthermore, 4D PC-VIPR was able to analyze the aortic FV at the same time, which is difficult with DUS, and we proved that the increase in FV in SMA was not due to the rise in cardiac output. The data from DUS and 4D PC-VIPR were significantly correlated, suggesting that 4D PC-VIPR could be useful for low-invasive assessment of SMA blood flow.

**Fig 7:**
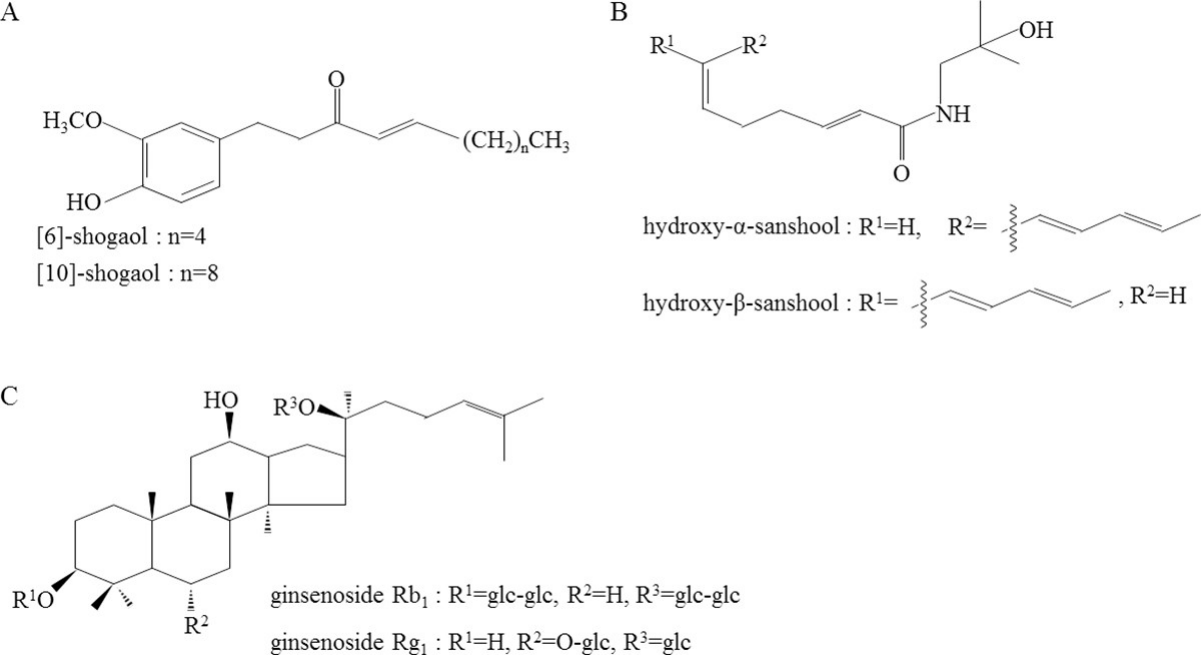
Structural formula of major components in TJ-100. (A) shogaol. (B) hydroxy-sanshool. (C) ginsenoside.

TJ-100 is indicated for the purpose of achieving relief from abdominal cold feeling and pain accompanied by abdominal flatulence. The usual dose is 15.0 g/d orally, divided into two or three doses before or between meals. In TJ-100, 43 components have been identified using liquid chromatography-tandem mass spectrometry analysis [39]. The major ingredients of TJ-100 are shogaols, hydroxy-sanshool, ginsenosides extracted from dried ginger, zanthoxylum fruit, and ginseng (Fig 7). Shogaols, hydroxy-sanshool, and gingerol are the agonists for TRPA1 and TRPV1, and the stimulation of these receptor is shown to increase the intestinal blood flow via the CGRP and ADM. CGRP is released by activating transient receptor potential cation channel subfamily V member 1 in the sensory nerve endings, and ADM is released by intestinal epithelial cells via the activation of transient receptor potential ankyrin 1 [20,28,29]. Following the administration of TJ-100, shogaol and hydroxy-sanshool reach the maximum concentration within 30 min and then gradually decreased [40]. Previous reports have shown that administering 5.0 g of TJ-100 increased SMA blood flow between 20 and 40 min by DUS [4]. In this study, 4D PC-VIPR or DUS was performed after 25 min after the administration of TJ-100 because the plasma concentration is higher than the effective level during this period and the increased SMA blood flow has been shown in previous reports [4,40]. The PC-VIPR examination requires about 15 min, which is comparable to the time for other techniques. Warming the abdomen is another well-used method to increase abdominal vessel flow, but it requires some materials placed in contact with the abdomen that could cause interference on MRI. Therefore, TJ-100, well-studied herbal medicine, that has a scientifically proven mechanism of action, was used in this study to increase the SMA blood flow.

As a characteristic feature of 4D PC-VIPR, it enables retrospective assessments of blood flow observed from arbitrary orientations even after a patient leaves the MR suite. Thus, US cannot repeat the measurements after the patient leaves the clinic. Furthermore, as recently reported by Sugiyama et al. [33], streamline analysis can be used to evaluate disturbed flow areas; thus, non-laminar flow can be avoided for the measurement plane placement. 4D PC-VIPR could also show 3D vascular morphology clearly without any contrast agents, which means that it is also useful even in patients with renal dysfunction or allergies to contrast agents.

In contrast, there are some disadvantages of 4D PC-VIPR. The major disadvantages are the extensive time required to shoot, lengthy evaluation time, and higher cost than DUS. Moreover, it has not yet been applied to mesenteric vessels in a clinical setting. Although 4D PC-VIPR was initially developed 15 y ago, thereafter, the technique has been exclusively studied at the University of Wisconsin–Madison. The technique was not commercially available at the time of writing this report. We believe that our report would enable a wider awareness regarding the usefulness of this technique.

In our data, the absolute value of the maximum flow velocity and FV had a discrepancy between DUS and 4D PC-VIPR. Both the methods record the highest velocities on a temporal basis; however, in the spatial basis, the data were different. The DUS detects the peak velocity at a single central point in the SMA. In contrast, the 4D PC-VIPR data showed the temporally highest velocity; however, it was averaged by a cross-sectional area. In the previous reports, the peak velocities [41,42] and FV [4] of SMA measured using Doppler ultrasonography was approximately 130 cm/s and 0.55 L/min, respectively. The peak cross-sectionally averaged velocity [33,43,44] and FV [45] of the SMA measured with 2D PC-MRI were about 80 cm/s and 0.30 L/min, respectively. These values were similar to those obtained in our study, suggesting that our data do not deviate from those of previous reports.

There were several limitations in this study that should be considered. The study was performed in a non-randomized fashion with a small sample size at a single institution. Further large-scale trials would be required to validate the usefulness of 4D PC-VIPR. In addition, the cost of 4D PC-VIPR is higher than that of any other technique and the time necessary for analysis may not be compatible with imaging of emergent patients. In two out of the 20 patients, the data was not generated by the MR scanner because of data storage overflow. The size of the row data for 4D PC-VIPR was > 3.0 GB, which was sometimes too large for the temporary data storage capacity of the clinical scanner. Regarding the sample size, the detected difference was similar to that of a previous study [4,46]; however, the numerical variation was larger than expected, and it is possible that a slightly larger sample size was required. However, it is important to note that there was a statistical difference even though the total number of required cases was small.

In conclusion, 4D PC-VIPR was found to be a useful technique for assessment of morphological and hemodynamic analysis of the SMA without using any contrast agent or ionizing radiation. Further studies using this new technique for assessment of the vascular flow direction of the abdominal arteries are needed.

## Supporting information

S1 Table Study subjects’ detailed information. Sex, age, measurement interval, last meal time, administration time, fasting duration, blood pressure, and body temperature of each subject for each examination.
(TIF)

## References

[1] Hirai K, Yoshinari D, Ogawa H, Nakazawa S, Takase Y, Tanaka K, et al Three-dimensional computed tomography for analyzing the vascular anatomy in laparoscopic surgery for right-sided colon cancer. Surg Laparosc Endosc Percutan Tech. 2013;23(6):536–9. Epub 2013/12/05. 10.1097/SLE.0b013e31828f66fb . 24300932

[2] Perko MJ. Duplex ultrasound for assessment of superior mesenteric artery blood flow. Eur J Vasc Endovasc Surg. 2001;21(2):106–17. Epub 2001/03/10. 10.1053/ejvs.2001.1313 . 11237782

[3] Nakamura T, Moriyasu F, Ban N, Nishida O, Tamada T, Kawasaki T, et al Quantitative measurement of abdominal arterial blood flow using image-directed Doppler ultrasonography: superior mesenteric, splenic, and common hepatic arterial blood flow in normal adults. J Clin Ultrasound. 1989;17(4):261–8. Epub 1989/05/01. 10.1002/jcu.1870170406 . 2523903

[4] Takayama S, Seki T, Watanabe M, Takashima S, Sugita N, Konno S, et al The effect of warming of the abdomen and of herbal medicine on superior mesenteric artery blood flow—a pilot study. Forsch Komplementmed. 2010;17(4):195–201. Epub 2010/09/11. 10.1159/000317845 . 20829597

[5] Frydrychowicz A, Landgraf BR, Niespodzany E, Verma RW, Roldán-Alzate A, Johnson KM, et al Four-dimensional velocity mapping of the hepatic and splanchnic vasculature with radial sampling at 3 tesla: a feasibility study in portal hypertension. J Magn Reson Imaging. 2011;34(3):577–84. Epub 2011/07/14. 10.1002/jmri.22712 21751287 PMC3417100

[6] Gu T, Korosec FR, Block WF, Fain SB, Turk Q, Lum D, et al PC VIPR: a high-speed 3D phase-contrast method for flow quantification and high-resolution angiography. AJNR Am J Neuroradiol. 2005;26(4):743–9. Epub 2005/04/09. . 15814915 PMC7977085

[7] Chang W, Loecher MW, Wu Y, Niemann DB, Ciske B, Aagaard-Kienitz B, et al Hemodynamic changes in patients with arteriovenous malformations assessed using high-resolution 3D radial phase-contrast MR angiography. AJNR Am J Neuroradiol. 2012;33(8):1565–72. Epub 2012/04/14. 10.3174/ajnr.A3010 22499844 PMC6278605

[8] Velikina JV, Johnson KM, Wu Y, Samsonov AA, Turski P, Mistretta CA. PC HYPR flow: a technique for rapid imaging of contrast dynamics. J Magn Reson Imaging. 2010;31(2):447–56. Epub 2010/01/26. 10.1002/jmri.22035 20099362 PMC2897749

[9] Ansari SA, Schnell S, Carroll T, Vakil P, Hurley MC, Wu C, et al Intracranial 4D flow MRI: toward individualized assessment of arteriovenous malformation hemodynamics and treatment-induced changes. AJNR Am J Neuroradiol. 2013;34(10):1922–8. Epub 2013/05/04. 10.3174/ajnr.A3537 . 23639564 PMC7965432

[10] Frydrychowicz A, Landgraf B, Wieben O, François CJ. Images in Cardiovascular Medicine. Scimitar syndrome: added value by isotropic flow-sensitive four-dimensional magnetic resonance imaging with PC-VIPR (phase-contrast vastly undersampled isotropic projection reconstruction). Circulation. 2010;121(23):e434–6. Epub 2010/06/16. 10.1161/CIRCULATIONAHA.109.931857 . 20547935 PMC11872175

[11] François CJ, Srinivasan S, Schiebler ML, Reeder SB, Niespodzany E, Landgraf BR, et al 4D cardiovascular magnetic resonance velocity mapping of alterations of right heart flow patterns and main pulmonary artery hemodynamics in tetralogy of Fallot. J Cardiovasc Magn Reson. 2012;14(1):16 Epub 2012/02/09. 10.1186/1532-429X-14-16 22313680 PMC3305663

[12] Ishikawa T, Takehara Y, Yamashita S, Iwashima S, Sugiyama M, Wakayama T, et al Hemodynamic assessment in a child with renovascular hypertension using time-resolved three-dimensional cine phase-contrast MRI. J Magn Reson Imaging. 2015;41(1):165–8. Epub 2014/03/13. 10.1002/jmri.24522 . 24615925

[13] Motoyama D, Ishii Y, Takehara Y, Sugiyama M, Yang W, Nasu H, et al Four-dimensional phase-contrast vastly undersampled isotropic projection reconstruction (4D PC-VIPR) MR evaluation of the renal arteries in transplant recipients: Preliminary results. J Magn Reson Imaging. 2017;46(2):595–603. Epub 2017/02/06. 10.1002/jmri.25607 . 28152259

[14] Manabe N, Camilleri M, Rao A, Wong BS, Burton D, Busciglio I, et al Effect of daikenchuto (TU-100) on gastrointestinal and colonic transit in humans. Am J Physiol Gastrointest Liver Physiol. 2010;298(6):G970–5. Epub 2010/04/10. 10.1152/ajpgi.00043.2010 . 20378829

[15] Endo S, Nishida T, Nishikawa K, Nakajima K, Hasegawa J, Kitagawa T, et al Dai-kenchu-to, a Chinese herbal medicine, improves stasis of patients with total gastrectomy and jejunal pouch interposition. Am J Surg. 2006;192(1):9–13. Epub 2006/06/14. 10.1016/j.amjsurg.2006.01.022 . 16769267

[16] Yoshikawa K, Shimada M, Wakabayashi G, Ishida K, Kaiho T, Kitagawa Y, et al Effect of Daikenchuto, a Traditional Japanese Herbal Medicine, after Total Gastrectomy for Gastric Cancer: A Multicenter, Randomized, Double-Blind, Placebo-Controlled, Phase II Trial. J Am Coll Surg. 2015;221(2):571–8. Epub 2015/07/05. 10.1016/j.jamcollsurg.2015.03.004 . 26141466

[17] Tokita Y, Satoh K, Sakaguchi M, Endoh Y, Mori I, Yuzurihara M, et al The preventive effect of Daikenchuto on postoperative adhesion-induced intestinal obstruction in rats. Inflammopharmacology. 2007;15(2):65–6. Epub 2007/04/24. 10.1007/s10787-006-1552-2 . 17450444

[18] Yoshikawa K, Shimada M, Nishioka M, Kurita N, Iwata T, Morimoto S, et al The effects of the Kampo medicine (Japanese herbal medicine) "Daikenchuto" on the surgical inflammatory response following laparoscopic colorectal resection. Surg Today. 2012;42(7):646–51. Epub 2011/12/29. 10.1007/s00595-011-0094-4 . 22202972

[19] Kono T, Kanematsu T, Kitajima M. Exodus of Kampo, traditional Japanese medicine, from the complementary and alternative medicines: is it time yet? Surgery. 2009;146(5):837–40. Epub 2009/09/12. 10.1016/j.surg.2009.06.012 . 19744449

[20] Kono T, Kaneko A, Omiya Y, Ohbuchi K, Ohno N, Yamamoto M. Epithelial transient receptor potential ankyrin 1 (TRPA1)-dependent adrenomedullin upregulates blood flow in rat small intestine. Am J Physiol Gastrointest Liver Physiol. 2013;304(4):G428–36. Epub 2013/01/01. 10.1152/ajpgi.00356.2012 23275609 PMC3566615

[21] Kikuchi D, Shibata C, Imoto H, Naitoh T, Miura K, Unno M. Intragastric Dai-Kenchu-To, a Japanese herbal medicine, stimulates colonic motility via transient receptor potential cation channel subfamily V member 1 in dogs. Tohoku J Exp Med. 2013;230(4):197–204. Epub 2013/07/31. 10.1620/tjem.230.197 . 23892797

[22] Nagano T, Itoh H, Takeyama M. Effect of Dai-kenchu-to on levels of 3 brain-gut peptides (motilin, gastrin and somatostatin) in human plasma. Biol Pharm Bull. 1999;22(10):1131–3. Epub 1999/11/05. 10.1248/bpb.22.1131 . 10549871

[23] Satoh K, Hayakawa T, Kase Y, Ishige A, Sasaki H, Nishikawa S, et al Mechanisms for contractile effect of Dai-kenchu-to in isolated guinea pig ileum. Dig Dis Sci. 2001;46(2):250–6. Epub 2001/04/03. 10.1023/a:1005636412287 . 11281170

[24] Shibata C, Sasaki I, Naito H, Ueno T, Matsuno S. The herbal medicine Dai-Kenchu-Tou stimulates upper gut motility through cholinergic and 5-hydroxytryptamine 3 receptors in conscious dogs. Surgery. 1999;126(5):918–24. Epub 1999/11/24. . 10568192

[25] Kono T, Kaneko A, Hira Y, Suzuki T, Chisato N, Ohtake N, et al Anti-colitis and -adhesion effects of daikenchuto via endogenous adrenomedullin enhancement in Crohn's disease mouse model. J Crohns Colitis. 2010;4(2):161–70. Epub 2010/12/03. 10.1016/j.crohns.2009.09.006 . 21122500

[26] Hayakawa T, Kase Y, Saito K, Hashimoto K, Ishige A, Komatsu Y, et al Effects of Dai-kenchu-to on intestinal obstruction following laparotomy. J Smooth Muscle Res. 1999;35(2):47–54. Epub 1999/08/27. 10.1540/jsmr.35.47 . 10463435

[27] Inoue A, Furukawa A, Yamamoto H, Ohta S, Linh NDH, Syerikjan T, et al Acceleration of small bowel motility after oral administration of dai-kenchu-to (TJ-100) assessed by cine magnetic resonance imaging. PLoS One. 2018;13(1):e0191044 Epub 2018/01/11. 10.1371/journal.pone.0191044 29320574 PMC5761958

[28] Jin XL, Shibata C, Naito H, Ueno T, Funayama Y, Fukushima K, et al Intraduodenal and intrajejunal administration of the herbal medicine, dai-kenchu-tou, stimulates small intestinal motility via cholinergic receptors in conscious dogs. Dig Dis Sci. 2001;46(6):1171–6. Epub 2001/06/21. 10.1023/a:1010690624187 . 11414290

[29] Kono T, Koseki T, Chiba S, Ebisawa Y, Chisato N, Iwamoto J, et al Colonic vascular conductance increased by Daikenchuto via calcitonin gene-related peptide and receptor-activity modifying protein 1. J Surg Res. 2008;150(1):78–84. Epub 2008/06/20. 10.1016/j.jss.2008.02.057 . 18561951

[30] Gill RW. Measurement of blood flow by ultrasound: accuracy and sources of error. Ultrasound Med Biol. 1985;11(4):625–41. Epub 1985/07/01. 10.1016/0301-5629(85)90035-3 . 2931884

[31] Van Bel F, Van Zwieten PH, Guit GL, Schipper J. Superior mesenteric artery blood flow velocity and estimated volume flow: duplex Doppler US study of preterm and term neonates. Radiology. 1990;174(1):165–9. Epub 1990/01/01. 10.1148/radiology.174.1.2403678 . 2403678

[32] Taylor KJ, Holland S. Doppler US. Part I. Basic principles, instrumentation, and pitfalls. Radiology. 1990;174(2):297–307. Epub 1990/02/01. 10.1148/radiology.174.2.2404309 . 2404309

[33] Sugiyama M, Takehara Y, Kawate M, Ooishi N, Terada M, Isoda H, et al Optimal Plane Selection for Measuring Post-prandial Blood Flow Increase within the Superior Mesenteric Artery: Analysis Using 4D Flow and Computational Fluid Dynamics. Magn Reson Med Sci. 2020 Epub 2020/02/06. 10.2463/mrms.mp.2019-0089 . 32009062 PMC7809144

[34] Tokita Y, Yuzurihara M, Sakaguchi M, Satoh K, Kase Y. The pharmacological effects of Daikenchuto, a traditional herbal medicine, on delayed gastrointestinal transit in rat postoperative ileus. J Pharmacol Sci. 2007;104(4):303–10. Epub 2007/08/02. 10.1254/jphs.fp0070831 . 17666868

[35] Nishi M, Shimada M, Uchiyama H, Ikegami T, Arakawa Y, Hanaoka J, et al The beneficial effects of Kampo medicine Dai-ken-chu-to after hepatic resection: a prospective randomized control study. Hepatogastroenterology. 2012;59(119):2290–4. Epub 2013/02/26. 10.5754/hge10115 . 23435143

[36] Ogasawara T, Morine Y, Ikemoto T, Imura S, Fujii M, Soejima Y, et al Influence of Dai-kenchu-to (DKT) on human portal blood flow. Hepatogastroenterology. 2008;55(82–83):574–7. Epub 2008/07/11. . 18613410

[37] Faul F, Erdfelder E, Buchner A, Lang AG. Statistical power analyses using G*Power 3.1: tests for correlation and regression analyses. Behav Res Methods. 2009;41(4):1149–60. Epub 2009/11/10. 10.3758/BRM.41.4.1149 . 19897823

[38] Faul F, Erdfelder E, Lang AG, Buchner A. G*Power 3: a flexible statistical power analysis program for the social, behavioral, and biomedical sciences. Behav Res Methods. 2007;39(2):175–91. Epub 2007/08/19. 10.3758/bf03193146 . 17695343

[39] Iwabu J, Watanabe J, Hirakura K, Ozaki Y, Hanazaki K. Profiling of the compounds absorbed in human plasma and urine after oral administration of a traditional Japanese (kampo) medicine, daikenchuto. Drug Metab Dispos. 2010;38(11):2040–8. Epub 2010/08/07. 10.1124/dmd.110.033589 . 20689019

[40] Munekage M, Kitagawa H, Ichikawa K, Watanabe J, Aoki K, Kono T, et al Pharmacokinetics of daikenchuto, a traditional Japanese medicine (kampo) after single oral administration to healthy Japanese volunteers. Drug Metab Dispos. 2011;39(10):1784–8. Epub 2011/07/05. 10.1124/dmd.111.040097 . 21724872

[41] Sjekavica I, Barbarić-Babić V, Krznarić Z, Molnar M, Cuković-Cavka S, Stern-Padovan R. Assessment of Crohn's disease activity by doppler ultrasound of superior mesenteric artery and mural arteries in thickened bowel wall: cross-sectional study. Croat Med J. 2007;48(6):822–30. Epub 2007/12/13. 10.3325/cmj.2007.6.822 18074417 PMC2213815

[42] Moneta GL, Yeager RA, Dalman R, Antonovic R, Hall LD, Porter JM. Duplex ultrasound criteria for diagnosis of splanchnic artery stenosis or occlusion. J Vasc Surg. 1991;14(4):511–8; discussion 8–20. Epub 1991/10/01. . 1920649

[43] Rose MJ, Jarvis K, Chowdhary V, Barker AJ, Allen BD, Robinson JD, et al Efficient method for volumetric assessment of peak blood flow velocity using 4D flow MRI. J Magn Reson Imaging. 2016;44(6):1673–82. Epub 2016/05/19. 10.1002/jmri.25305 27192153 PMC5115994

[44] Siedek F, Giese D, Weiss K, Ekdawi S, Brinkmann S, Schroeder W, et al 4D flow MRI for the analysis of celiac trunk and mesenteric artery stenoses. Magn Reson Imaging. 2018;53:52–62. Epub 2018/07/17. 10.1016/j.mri.2018.06.021 . 30008436

[45] Totman JJ, Marciani L, Foley S, Campbell E, Hoad CL, Macdonald IA, et al Characterization of the time course of the superior mesenteric, abdominal aorta, internal carotid and vertebral arteries blood flow response to the oral glucose challenge test using magnetic resonance imaging. Physiol Meas. 2009;30(10):1117–36. Epub 2009/09/18. 10.1088/0967-3334/30/10/011 . 19759401

[46] Takayama S, Seki T, Watanabe M, Monma Y, Sugita N, Konno S, et al The herbal medicine Daikenchuto increases blood flow in the superior mesenteric artery. Tohoku J Exp Med. 2009;219(4):319–30. Epub 2009/12/08. 10.1620/tjem.219.319 . 19966532

