# Supplementary figures and images for: Daikenchuto increases blood flow in the superior mesenteric artery in humans: A comparison study between four-dimensional phase-contrast vastly undersampled isotropic projection reconstruction magnetic resonance imaging and Doppler ultrasound
Source: PLoS One. 2021 Jan 27;16(1):e0245878. doi: 10.1371/journal.pone.0245878 (PMC7840032; doi:10.1371/journal.pone.0245878)

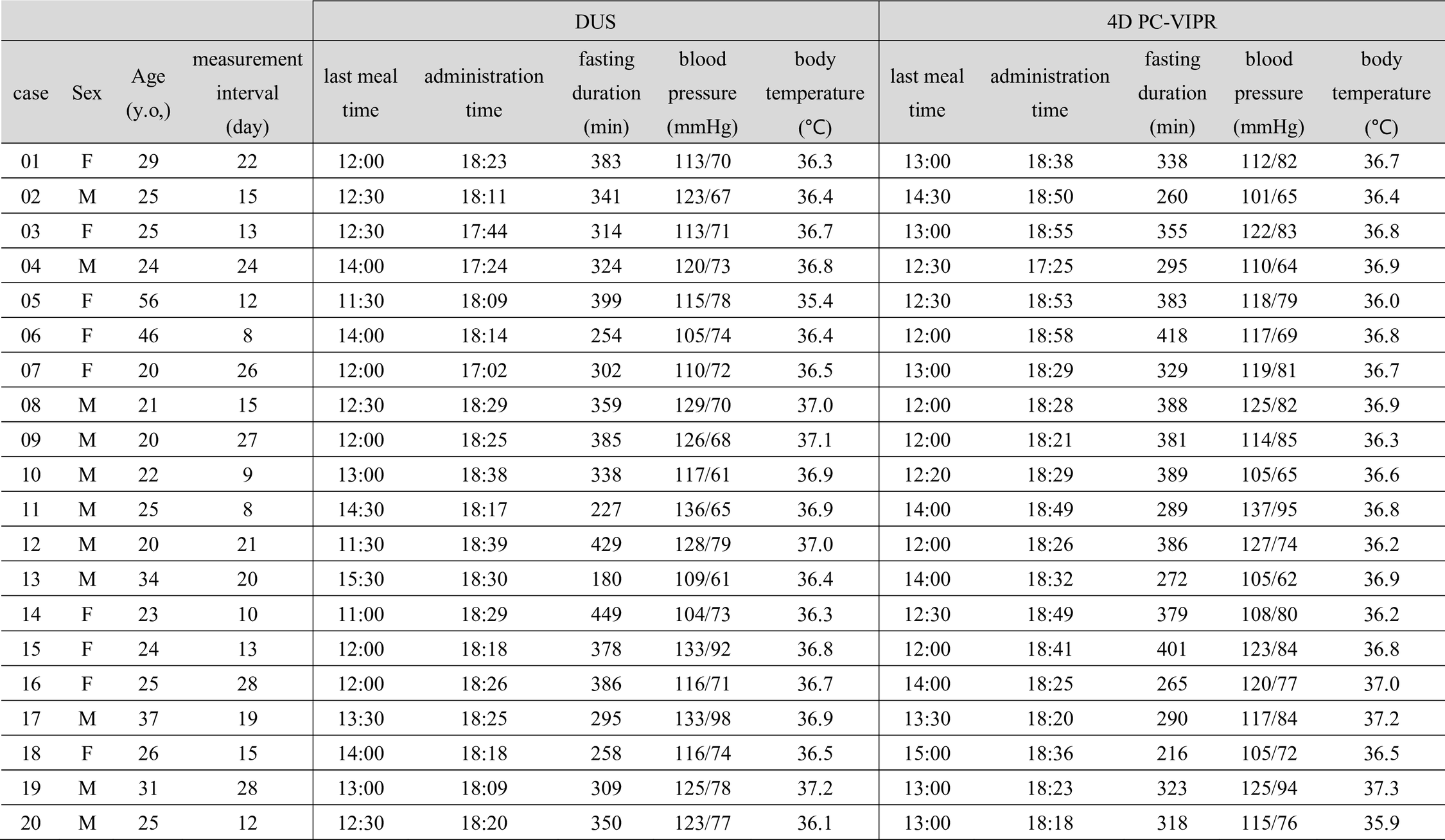

Supplement: S1 Table — Sex, age, measurement interval, last meal time, administration time, fasting duration, blood pressure, and body temperature of each subject for each examination. (TIF) [file pone.0245878.s001.tif]
